# Composite Structure as a Stress Wave Barrier Zone Under Impulse Loading: Microscale Numerical Analysis of Attenuation

**DOI:** 10.3390/ma18245599

**Published:** 2025-12-12

**Authors:** Zuzana Murčinková, Dominik Sabol, Petr Baron

**Affiliations:** Faculty of Manufacturing Technologies, Technical University of Košice, 080 01 Prešov, Slovakia; zuzana.murcinkova@tuke.sk (Z.M.); dominik.sabol@tuke.sk (D.S.)

**Keywords:** discontinuously reinforced composites, microscale, stress-wave, scattering, attenuation, explicit finite element analysis, impulse loading

## Abstract

**Highlights:**

**What are the main findings?**

- In discontinuously reinforced composites, hollow inclusions enhance stress wave attenuation by over 20% compared to solid ones due to greater deformation and scattering.
- Elongated inclusion orientation strongly affects attenuation, with perpendicular alignment increasing efficiency by 18.5%.
- A compliant interlayer and specified inclusion distribution further improve attenuation by 3–11%.

**What is the implication of the main finding?**

- Optimizing inclusion shape and orientation enhances stress wave attenuation.
- Hollow inclusions and compliant large interlayers increases energy dissipation and scattering.
- Controlled inclusion distribution enables efficient stress wave barrier zones design.

**Abstract:**

This study investigates the design factors of stress wave barrier zones intended for manufacturing machines under impulse loading, using polymer discontinuously reinforced composites with specified internal microstructures, which effectively suppress stress at the wave front, promote uniform stress distribution, improve impact resistance, and reduce vibrations and noise. Two-dimensional representative unit cells and explicit finite element simulations were used to analyze stress wave propagation under impulse loading. The effects of inclusion shape, orientation, distribution, interlayer, and size of the interface on stress wave scattering and attenuation were examined. In our models, hollow inclusions demonstrated 20.6% higher attenuation compared to solid inclusions, with the hollow fiber inclusion showing the most significant improvement. Inclusion orientation relative to the stress wave direction affected attenuation by 18.5%, while redistribution of inclusions and addition of a compliant interlayer contributed additional increments of 3–11%. These results highlight the critical role of microscale topology in stress barrier zone designing, such that the combined adjustment of inclusion shape, orientation, interlayer presence, and spatial distribution provides an effective strategy to maximize stress wave attenuation.

## 1. Introduction

In modern manufacturing and technological systems, mechanical components are increasingly designed to operate at higher speeds, power levels, and loading frequencies. This trend results in elevated dynamic stresses within structures, often manifested in undesirable vibrations and increased deformability of mechanical systems. Excessive oscillations negatively affect the accuracy of manufacturing processes, the operational reliability, and the service life of both individual components and entire devices.

During the operation of machines and equipment, impulsive forces (e.g., sudden braking) may occur, which can multiply the stresses within the structure during operation—a highly unfavorable phenomenon. Impulsive and impactful forces can also originate from vibrations of a random nature, causing stochastic shocks. Prolonged exposure to such impact vibrations leads to excessive wear and progressive material degradation.

The concept of stress barrier zones is designed to attenuate, trap, or redirect the propagation of stress waves. These zones are primarily used to protect structures and people from the effects of vibrations—such as those induced by transportation systems—as well as explosions or seismic events, across sectors including mining, constructions, energy, defense, and civil infrastructures. In mechanical engineering, the application of stress barrier zones is gaining momentum, particularly in connection with metamaterials and other advanced structural isolation components that are being actively developed to enhance vibration mitigation and structural resilience.

To mitigate stress wave effects, lattice-structured materials, honeycombs, 3D metamaterials, and auxetic materials are used, primarily due to their distinctive geometries rather than material composition. However, their design and fabrication are costly and technologically demanding, requiring specialized designing methods, technologies, materials, and complex geometries. Thus, a practical strategy to break the limitations of the aforementioned materials is the implementation of polymer-based discontinuously reinforced composites in stress barrier zones, offering reduced material and manufacturing demands while maintaining effective wave mitigation properties.

While composites are typically recognized for enhancing stiffness and strength, their damping properties should not be overlooked. In discontinuously reinforced composites, inclusions (particles, short fibers) are generally considered to be the reinforcing phase, but their contribution also lies in damping.

In our previous research on mitigating vibration amplitudes in manufacturing equipment [1,2,3], we demonstrated the benefits of redesigning components using discontinuously reinforced polymer and multi-layered composites applied into/onto existing component cavities and surfaces. Notably, significant amplitude reductions were observed mainly within the resonance frequency range. To better understand the microscale mechanisms underlying vibration attenuation in these composites, we numerically analyzed stress wave propagation within a representative micro-unit of the composite’s repeating structure. Using an explicit finite element approach designed for rapid dynamic events, such as impulse loading, we simulated the propagation of stress waves within the discontinuously reinforced composite microstructure.

Works [4,5,6,7,8,9] share a common focus on reducing vibrations and improving the dynamic response of complex composite systems through appropriate modification of their internal structure. A study [4] demonstrated that in 3D-printed composites arranging aramid and glass fiber reinforcements at angles of 45°, 90°, and ±45° significantly enhances damping capacity, whereas the orientation of carbon fibers at 0° or 0°/90° reduces damping. Another study [5] further showed that epoxy–cement composites with an optimal amount of nano SiO_2_ and graphene exhibit 92% higher compressive strength and 38% higher damping factor than conventional cement mixtures. A combined numerical and experimental investigation in [6] examined the vibration response of additively manufactured PLA (polylactic acid) composites with fiber reinforcement, finding that the 0–0° orientation provides the highest natural frequencies and, when combined with MFC (macro-fiber composite) piezoelectric actuators, significantly enhances active damping. In the field of high-speed rotating machinery, another study [7] demonstrated that integrating composites filled with carbon powder, hollow glass microspheres, and sand into the bearing housings can reduce the resonance peak by 30–70%, confirming the beneficial impact of composite fillers on dynamic stability. All of these studies demonstrate that the appropriate combination of reinforcement materials, nanoparticles, and design modifications can effectively suppress resonances and ensure improved dynamic response in composite systems. Additionally, optimizing the microstructure of a composite material can enhance its dynamic properties without significantly increasing weight or macrostructural complexity [8]. Another study [8] stated that the optimal heterogeneous distribution of the core in a structural layered sandwich composite can nearly triple its stability compared to a homogeneous distribution under static loading.

Material damping and stress wave attenuation are closely related but have distinct phenomena. While stress wave attenuation in homogeneous materials is largely proportional to material damping (in [10]), in heterogeneous composites, wave scattering also contributes significantly (in [11,12,13]). In our presented study, alongside visualizing stress wave propagation, we analyze structural features that enhance scattering, thereby slowing and facilitating early “trapping” of stress waves within the composite. Regions exhibiting increased damping and scattering can be defined as stress wave barrier zones. By tailoring the composite structure, the propagation direction and speed of stress waves can also be controlled. The presence of such barrier zones is particularly beneficial under impulse loading, enabling rapid suppression of stress concentrations at the wave-front, uniform stress distribution, improved impact resistance, and reduced vibrations and noise.

To analyze stress wave scattering as an indirect contribution to damping, studying stress wave propagation within a material micro-unit using the explicit finite element method proves to be a highly suitable method. The authors of work [14] describe the development of the explicit D-FE2 method, which enables simultaneous modeling of macro- and microscales in dynamic problems, including stress wave propagation in porous materials, impact testing of honeycomb structures, and damage in fiber-reinforced composites.

From previous research on material damping in polymer-based particulate composites, including those with discontinuous fibers, it is well established that one of the primary sources of damping is the viscoelasticity of the polymer matrix [15,16], in which the behavior of molecular chains during deformation leads to the dissipation of mechanical energy and its conversion into heat. According to Bendat and Piersol [15], the primary macroscopic manifestation of damping in structural materials is the dissipation, or loss, of mechanical energy in vibrating elements of a mechanical system. The presence and intensity of damping can be quantified using a hysteresis loop (in [16]), which allows the determination of the amount of mechanical energy lost from the system. This approach is typical for homogeneous viscoelastic or anelastic materials, where energy is converted into heat during cyclic loading.

Another source of energy dissipation, as reported in the literature, is micro-slipping, interfacial friction, and pull-out/de-bonding [17,18,19,20] at the matrix–reinforcement interface, as well as the local opening and closing of microcracks [21,22]. Less explored mechanisms of energy dissipation in polymer particulate composites include the effects of elastic/plastic deformation of particles [22,23,24], particle–particle friction in highly filled polymer systems [20,25], and particle resonance [26,27]. A related research area concerns the interfacial transition zone [26,28], as it is generally challenging to combine increased damping while simultaneously enhancing strength through interfacial mechanisms. In [28,29], a strategy for “interface design” is proposed by modifying the interfacial transition zone using a nanocomposite coating.

In this study, we did not aim to examine the effects of material mechanical properties, such as material density *ρ*, elastic modulus *E*, shear modulus *G*, Poisson’s ratio *ν*, and their matrix/inclusion ratios, nor the influence of chemical composition, additional heterogeneities, or inclusion size, as these topics have already been extensively addressed in the literature, e.g., in [30,31,32,33,34,35]. In [36], the authors investigated the effects of fiber volume fraction, layer thickness, and reinforcement orientation on damping ratio and dynamic response under sinusoidal excitation. The study [36] also demonstrates that increasing the reinforcement fraction and applying appropriate layer sequencing can effectively influence damping.

In line with current research trends, this study focuses on defining the design parameters necessary for implementing a stress wave barrier zone in existing manufacturing machines by incorporating a polymer discontinuously reinforced composite with a specified internal microstructure. This structure enhances stress wave scattering, which indirectly contributes to the overall damping effect. The analysis of parameters required to create a stress wave barrier zone against the harmful effects of impulse loading relies primarily on the geometric parameters of the composite’s internal architecture—such as inclusion shape, inclusion orientation (with respect to the dominant direction of impulse loading), the presence of an interlayer, and the influence of inclusion arrangement for a constant inclusion fraction.

## 2. Methodology, Materials, and Numerical Models

The microscale analysis of discontinuously reinforced composites was conducted in Abaqus/CAE 2022 (Dassault Systèmes SIMULIA, Johnston, RI, USA). The matrix material was reinforced with discrete inclusions of various shapes (circular, elliptical, rectangular, and hollow variants). Moreover, the models with a third interface layer were analyzed. These inclusions were oriented either parallel or perpendicular to the direction of the impulse loading. Material mechanical properties of the matrix, inclusions (particles/fibers), and interlayer, if included in model, are in Table 1.

To analyze the dynamic response under impact loading, two-dimensional (2D) models of representative unit cells (RUCs) were created to capture the repeating microgeometry of the composite. Using the explicit finite element analysis, the propagation of a stress wave induced by an impulse load was simulated, and the time histories of Mises stress were evaluated at monitoring points. Attenuation was quantified based on the rate of amplitude decay of this stress wave.

The computational model assumed that each phase of the composite behaves as a homogeneous, isotropic, and ideally elastic material. The material response was considered within the linear stress–strain range, corresponding to elastic wave propagation without plastic deformation or shock waves. Perfect bonding between inclusions and the matrix was assumed, with no interfacial slip, and the RUC was considered free of residual stresses.

**Figure 1:**
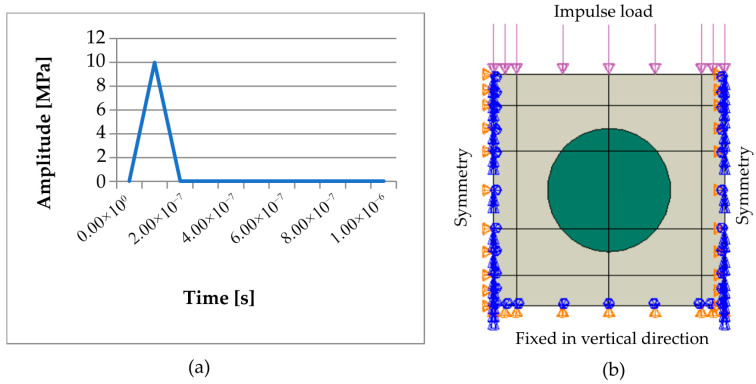
(**a**) Impulse load, (**b**) boundary conditions, and model geometry [37].

The impulse load (Figure 1a) was modeled as a pressure pulse acting perpendicular to the top edge of the model (Figure 1b). The pressure increased linearly from 0 MPa to 10 MPa and then returned to zero over 2 × 10^−7^ s, creating a short but intense stress impulse.

The degree of freedom in the vertical direction was fixed at the bottom edge of the numerical model, and mirror symmetry boundary conditions were applied on both vertical edges (Figure 1b). The simulation was performed in 100 steps, with the total time interval of 1 × 10^−5^ s evenly divided into increments of 1 × 10^−7^ s. Under this setup, the time increment was just below the stability limit of the explicit integration, so the simulation maintained numerical stability with a sufficiently small time step to accurately capture the stress wave propagation.

**Figure 2:**
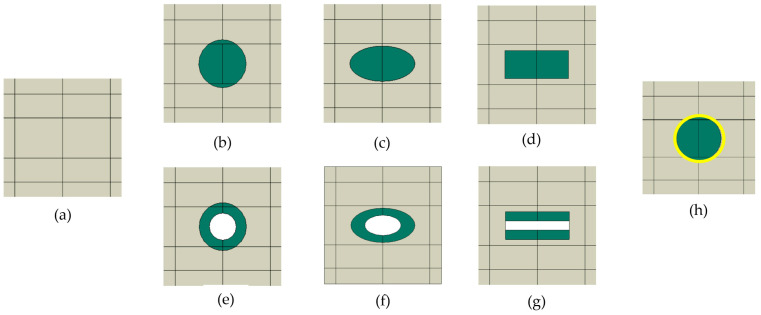
RUCs with different inclusion shapes: (**a**) RUC_0_, i.e., inclusion-free model; (**b**) RUC_circle_; (**c**) RUC_ellipse_; (**d**) RUC_rectangle_; (**e**) RUC_hollow circle_; (**f**) RUC_hollow ellipse_; (**g**) RUC_hollow rectangle_; (**h**) RUC_interlayer_.

The loading conditions and boundary constraints in our study result primarily in in-plane deformation. Under such conditions, a 2D approximation provides a reasonable representation of the relevant stress and strain fields. Figure 2 shows the individual numerical models of RUCs with different shapes of the inclusions. The 2D cross-sectional geometries (circle, rectangle, ellipse, etc.) are idealized representations of inclusions (reinforcement), which can correspond to fibers in fiber-reinforced composites or particles in particle-reinforced composites. Depending on the composite type, these inclusion shapes may represent either particles or fibers. In this sense, the geometries serve as simplified, generic inclusions to study the mechanical behavior of different types of discontinuously reinforced composites.

**Figure 3:**
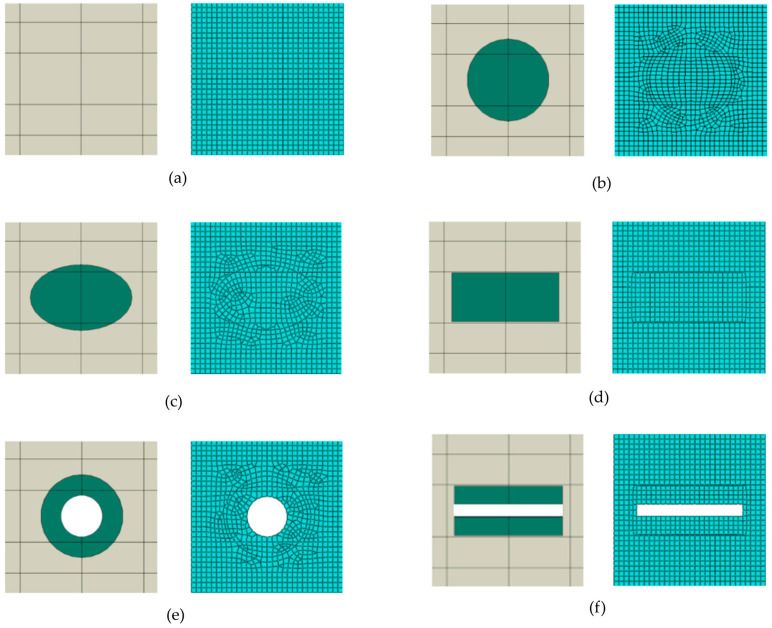
Numerical models with finite element mesh (CPS4R): (**a**) RUC_0_; (**b**) RUC_circle_; (**c**) RUC_ellipse_; (**d**) RUC_rectangle_; (**e**) RUC_hollow circle_; (**f**) RUC_hollow rectangle_.

The RUC_interlayer_ introduces a transition zone as a thin interphase layer between the stiffer inclusion and the softer matrix (Figure 2h). All RUCs containing an inclusion were compared with a reference model without a reinforcement, RUC_0_, (Figure 2a and Figure 3a), i.e., an empty homogeneous matrix. In all numerical models, the same matrix-to-inclusion area ratio (*A*_m_:*A*_inc_) was maintained to ensure consistency across the different models.

The finite element mesh (Figure 3) for RUCs was composed of four-node bilinear quadrilateral plane stress elements with reduced integration (CPS4R). The mesh density was set to 0.55 mm with a minimum curvature factor of 0.1 (10%), resulting in, for example, 1178 elements in RUC_circle_ in Figure 3b.

In presented numerical models, both the matrix and the inclusions were considered ideally elastic, meaning that an ideally elastic material acts as a perfect mechanical energy accumulator with no dissipation, and thus material damping does not exist. However, the advantage of our study lies in its analysis of the stress wave attenuation in discontinuously reinforced composites as the cumulative effect of the different phenomena, which influence damping and indirectly enhance the resulting attenuation, with the aim of controlling the wave.

The phenomena involved in stress wave attenuation is scattering caused solely by wave interaction at the matrix–inclusion interfaces. Upon wave incidence on an interface, interactions depend on differences in material properties (density, wave speed, impedance). The wave encounters heterogeneities, and energy is not dissipated but redistributed—scattered in multiple directions—resulting in a reduction in amplitude at stress wave-front, i.e., attenuation of the original stress wave. Scattering involves the following phenomena:

- Reflection—a portion of the wave energy is reflected back;
- Transmission and subsequent refraction—part of the wave energy enters the inclusion, changing its direction and amplitude;
- Interference of reflected waves—superposition of waves.

The stress wave attenuation can be expressed as follows:

(1) $$\alpha_{attenuation}=\alpha_{reflexion}\;+\;\alpha_{transmition}\;+\;\alpha_{refraction}\;+\;\alpha_{interference}$$

Then the overall material damping can be expressed as follows:

(2) $$\alpha_{overall\;damping}=\alpha_{dissipation}\;+\;\alpha_{attenuation}$$

Considering the actual material properties of the matrix and inclusions (including interfaces), energy dissipation occurs, resulting in irreversible consumption of energy. Mechanical energy is converted into heat due to anelasticity of the material itself, i.e., microplastic deformations and random microstructural defects, such as dislocations or microcracks. This energy loss is typically modeled using a hysteresis loop, in which the damping force is proportional to the velocity and the natural frequency of vibration. The energy loss in a loaded material system during a single cycle can be expressed using a line integral:

(3) $$\Delta W_{d}=\oint \sigma d\varepsilon$$

where Δ*W_d_* is the work dissipated per cycle under cyclic loading (corresponding to the area of the hysteresis loop), σ is the stress, and ε is the strain developed in the loaded system. The energy loss coefficient, or the relative damping factor *Ψ*, is defined as the ratio of the area of the hysteresis loop (representing the energy loss Δ*W_d_* during one cycle) to the maximum elastic strain energy *W* stored in the material during the same cycle.

(4) $$\Psi =\frac{\Delta W_{d}}{W}$$

**Figure 4:**
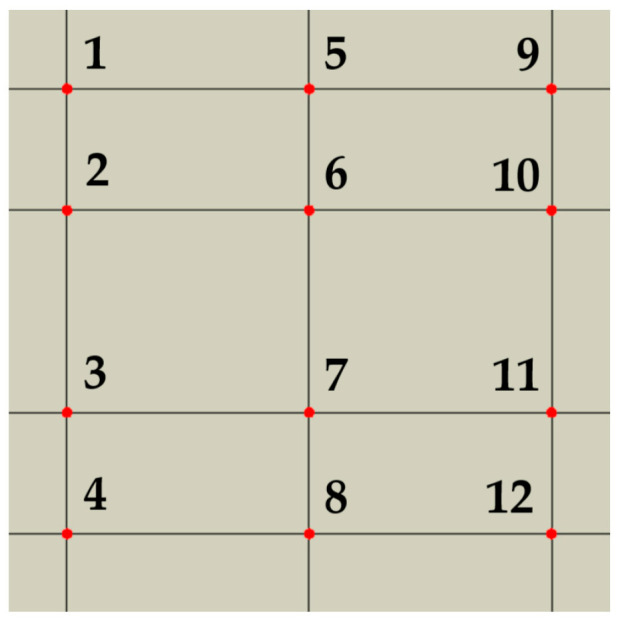
Numerical model showing the location of monitoring points for stress wave attenuation evaluation [37].

As mentioned above, the impulse pressure load applied to the top edge of the model generated a stress wave that propagated through the composite structure. Based on the values at the wave front, the stress wave attenuation was calculated along three lines of monitoring points: 1–4, 5–8, and 9–12 (Figure 4). The stress wave attenuation along each line of points is determined as follows:

(5) $$\alpha_{attenuation}=\frac{\sigma_{Mi}-\sigma_{Mi\;+\;3}}{\sigma_{Mi}}\times 100\%;\;\;\;i=1,\;5,\;9,$$

where *σ*_Mi_ is the maximum amplitude value of the Mises stress at the wave-front of the propagating stress wave at the i-th monitoring point over time interval. The overall attenuation was determined as the average of the attenuations along lines 1–4, 5–8, and 9–12.

## 3. Numerical Results: Influence of Microstructural Factors on Stress Wave Scattering

**Figure 5:**
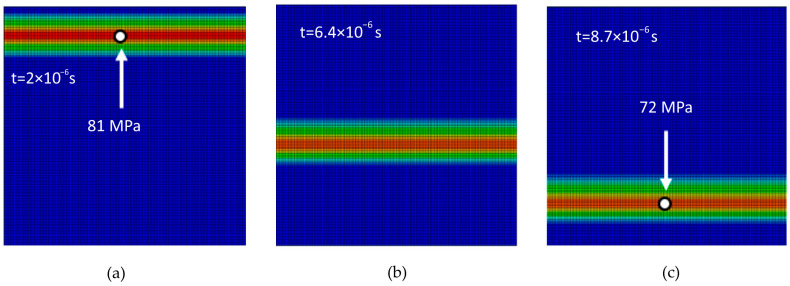
Stress wave propagation phases in the inclusion-free model RUC_0_: (**a**) phase 1; (**b**) phase 2; (**c**) phase 3.

For comparison, Figure 5 shows the stress wave propagation phases for the inclusion-free simulation model, RUC_0_. Wave propagation is not affected by any interaction with stiffer reinforcing material or interfaces. The stress wave remains homogeneous in homogeneous matrix. The RUC_0_ without an inclusion exhibits an attenuation of 12.5%.

### 3.1. Visualization of Stress Wave Propagation and Its Scattering in RUCs of Discontinuously Reinforced Composites

**Figure 6:**
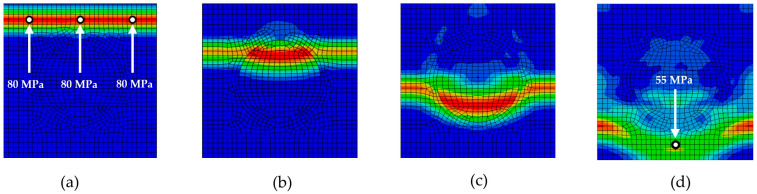
Stress wave propagation phases for RUC_circle_: (**a**) phase 1; (**b**) phase 2; (**c**) phase 3; (**d**) phase 4.

The application of an impulse load to the top edge of the discontinuously reinforced composite initiates the propagation of a stress wave in the vertical direction across the entire width of the model (Figure 6a). In the initial phase, the wave propagates without interacting with the reinforcing elements until it first contacts an inclusion. As shown in Figure 6b, upon reaching a stiffer inclusion, an interaction occurs between the wave and the inclusion, leading to a gradual reduction in stress amplitude (Figure 6c). As the wave continues to propagate through other regions of the composite material, a significant attenuation of the Mises stress at the wave-front is observed, with lower stress values recorded at the final monitoring points (Figure 6d).

**Figure 7:**
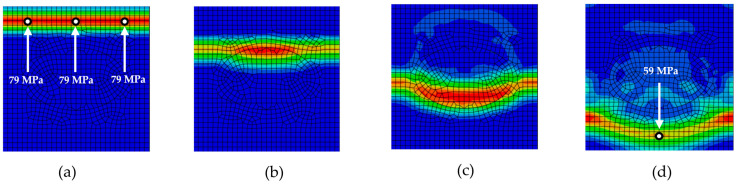
Stress wave propagation phases for RUC_ellipse_: (**a**) phase 1; (**b**) phase 2; (**c**) phase 3; (**d**) phase 4.

**Figure 8:**
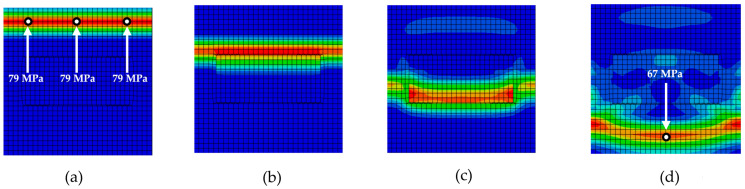
Stress wave propagation phases for RUC_rectangle_: (**a**) phase 1; (**b**) phase 2; (**c**) phase 3; (**d**) phase 4.

**Figure 9:**
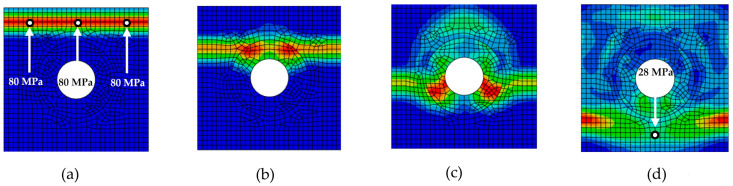
Stress wave propagation phases for RUC_hollow circle_: (**a**) phase 1; (**b**) phase 2; (**c**) phase 3; (**d**) phase 4.

**Figure 10:**
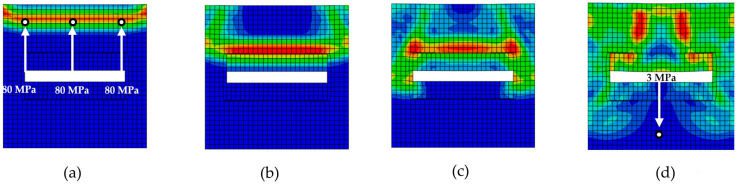
Stress wave propagation phases for RUC_hollow rectangle_: (**a**) phase 1; (**b**) phase 2; (**c**) phase 3; (**d**) phase 4.

Figure 6, Figure 7, Figure 8, Figure 9 and Figure 10 illustrate the various phases of stress wave propagation through the composite structure with different inclusion geometries and Mises stress values in monitoring points 1. 5, 8, and 9 in Figure 4.

**Figure 11:**
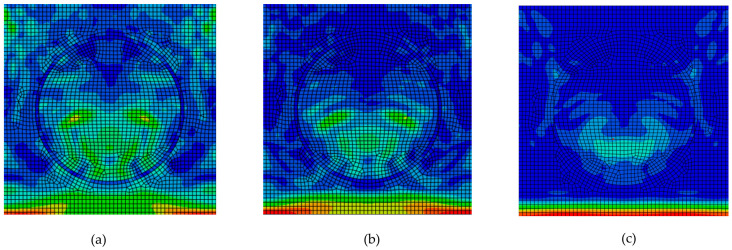
Phase 4 for RUC_interlayer_: (**a**) *E*_m_:*E*_interlayer_ = 1:0.2; (**b**) *E*_m_:*E*_interlayer_ = 1:0.5; (**c**) *E*_m_:*E*_interlayer_ = 1:1.5.

The influence of the interlayer on stress wave propagation depends on its properties. The interlayer can be stiffer or softer than the matrix, with ratios of *E*_m_:*E*_interlayer_ = 1:0.2, 1:0.5, and 1:1.5. The final propagation phase can be compared for all three ratios (Figure 11). For a softer interlayer (Figure 11a,b), the stress wave exhibits significantly greater scattering with visible numerous interactions, whereas a stiffer interlayer than the matrix results in only minor scattering (Figure 11c). The significant differences in scattering, including reflection, transmission, refraction, and interference phenomena shown in Figure 11, affect the magnitude of attenuation, which is quantified in Figure 14.

### 3.2. Inclusion Shape Factor

The results clearly demonstrate that the shape of the reinforcing inclusions has a significant impact on the stress wave attenuation in discontinuously reinforced composites. RUC models with hollow inclusions consistently exhibited higher attenuation compared to their solid counterparts, which can be attributed to more effective mechanical energy scattering due to different inclusion stiffness, internal structure, and increased interface.

**Figure 12:**
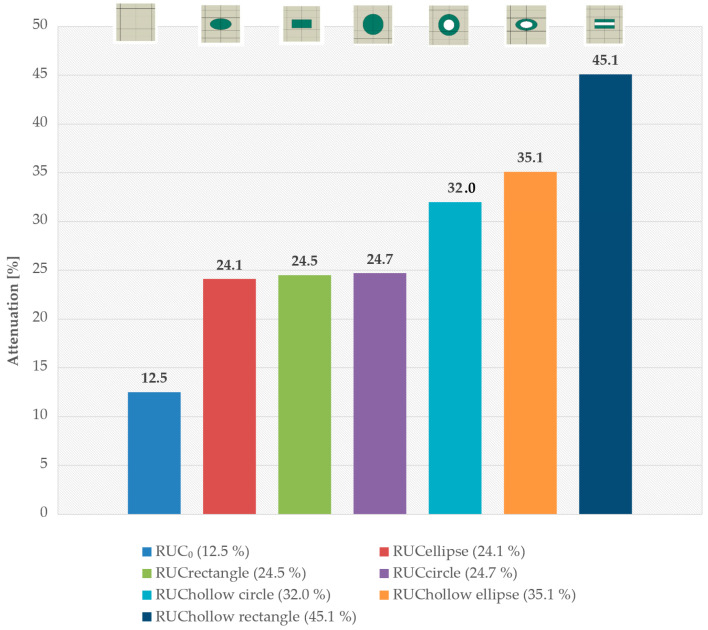
Stress wave attenuation for different inclusion shapes.

The highest attenuation among the models with solid inclusions was observed for the RUC_circle_, where the attenuation reached 24.7% (Figure 12). Compared to the reference model without inclusions, RUC_0_, the attenuation with solid inclusions was nearly twice as high. Models with hollow inclusions demonstrated that the achieved attenuation values exceeded those of the solid inclusion models. The hollow circular inclusion, RUC_hollow circle_, exhibited 7.3% higher attenuation than the solid circular inclusion, RUC_circle_. The hollow elliptical inclusion, RUC_hollow ellipse_, showed an 11.0% increase in attenuation compared to the solid elliptical inclusion. The most notable improvement was observed for the hollow rectangular inclusion (hollow fiber), RUC_hollow rectangle_, which achieved 20.6% higher attenuation than its solid equivalent, RUC_rectangle_.

By comparing the attenuation of RUCs with solid and hollow inclusions, it is indicated that the hollow core results in greater deformation, and consequently, the energy store of the wave is higher. This finding aligns with the assumption that shape anisotropies and internal cavities enhance the interaction between the wave and the reinforcing elements, which is suitable for a stress barrier zone. Deformation-based damping is a less well-known mechanism that significantly influences stress wave attenuation in composites containing hollow or softer inclusions as the matrix. In the case of solid inclusions, the stress wave primarily deforms the matrix during propagation, whereas in hollow inclusions, the inclusions themselves undergo substantial deformation. Furthermore, under significant deformation of a hollow inclusion, micro-slipping at the interface may occur, enhancing damping (this phenomenon is not included in the numerical models, which assume a perfectly bonded matrix–inclusion interface).

### 3.3. Inclusion Orientation Factor

**Figure 13:**
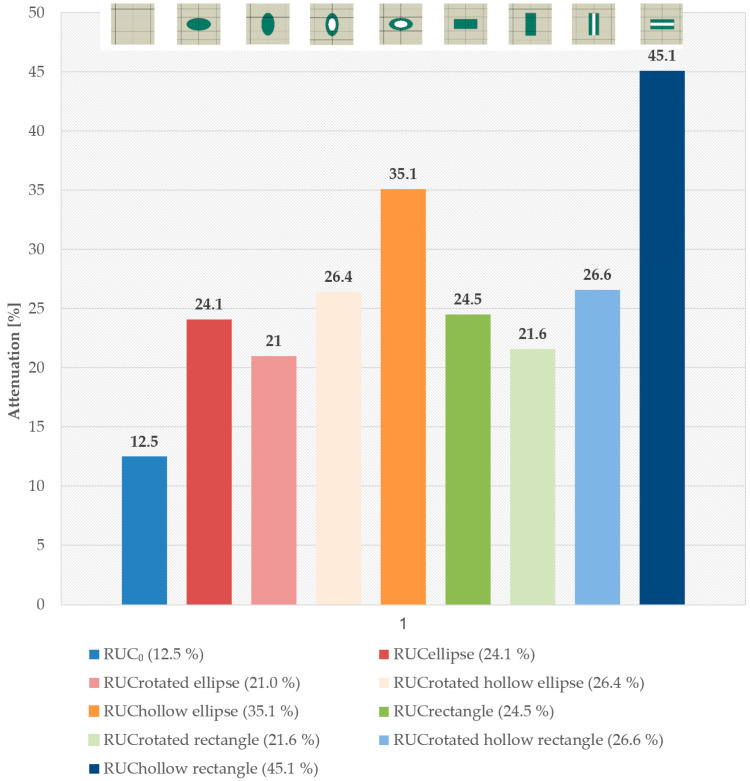
Stress wave attenuation for inclusions aligned parallel and perpendicular to wave propagation direction.

A numerical simulation was also performed for change in inclusion orientation relative to the direction of wave propagation. The results of the stress wave attenuation for inclusions rotated by 90° clearly show that inclusion orientation significantly affects the attenuation level (Figure 13). When the solid elliptical inclusion, RUC_ellipse_, with main axis perpendicular to the stress wave propagation (Figure 2c), was re-oriented by a 90°rotation, the total attenuation decreased by 3.1%, whereas for the hollow elliptical inclusion, RUC_hollow ellipse_, it dropped by 8.7%. With rotation of the inclusion’s principal axis of the solid rectangular inclusion, RUC_rectangle_, attenuation decreased by 2.9%, and for the rectangular inclusion with a longitudinal cavity, RUC_hollow rectangle_, the decrease reached 18.5%.

The elongated shape of the inclusions introduces shape anisotropy, making the mechanical impedance direction-dependent. The effects of inclusion orientation relative to wave propagation direction are as follows:

- Perpendicular orientation: The wave impacts the lateral surface of the elongated inclusion, which presents a sudden change in mechanical impedance due to shape anisotropy of inclusion. This abrupt impedance mismatch causes strong reflection and generates secondary waves that interfere and scatter in multiple directions. The enhanced scattering and energy redistribution increase stress wave attenuation and create an effective stress barrier zone. Additionally, the concentration of stress at the inclusion interfaces can lead to local high-stress gradients. The shape anisotropy of inclusions makes mechanical impedance direction-dependent.
- Parallel orientation: The inclusion aligns with wave propagation, presenting a smoother path with minimal impedance variation. Consequently, the wave travels more continuously, with weak reflection and low scattering, resulting in reduced stress wave attenuation. The directional dependence of these effects directly arises from the anisotropic inclusion shape and its influence on the local wave–material interactions.

### 3.4. Interlayer Presence Factor

**Figure 14:**
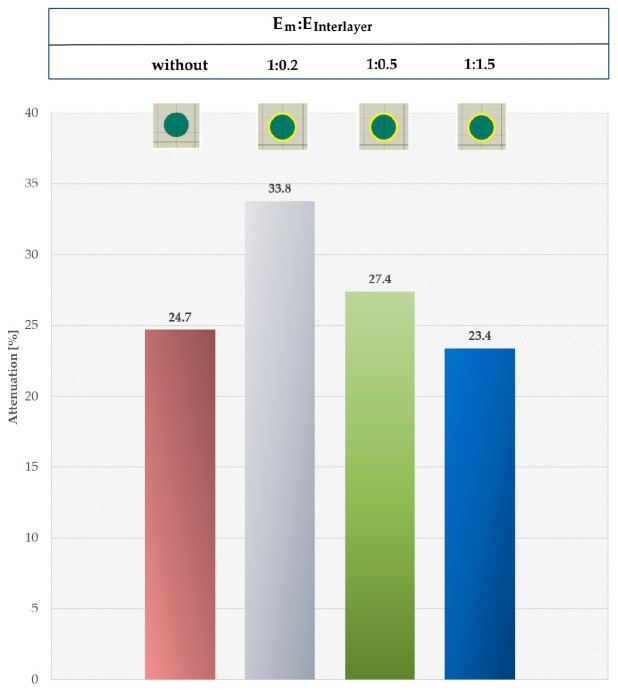
Comparison of the interlayer presence effect.

Although the interlayer in real reinforced composites is thin—on the order of micrometers to nanometers—it changes the mechanical properties and affects damping-related processes. A softer interlayer (*E*_m_:*E*_interlayer_ 1:0.2; 1:0.5, Figure 14) increases the stress wave attenuation of 9.1% and 2.7%, respectively, for the same interlayer thickness. A stiffer layer than matrix (*E*_m_:*E*_interlayer_ 1:1.5, Figure 14) lowers the stress wave attenuation. It can be expected that a thicker, softer interlayer will further increase the damping.

### 3.5. Inclusion Distribution and Interface Length Factor

**Figure 15:**
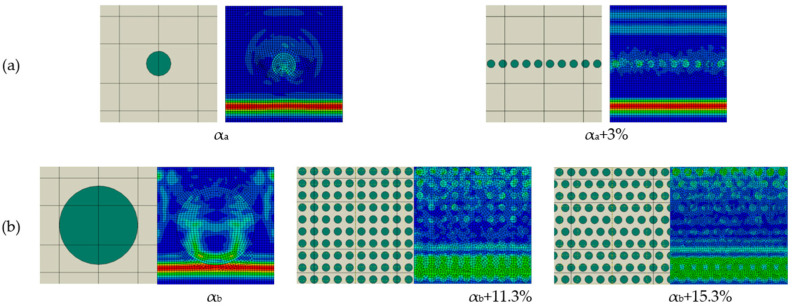
Effect of inclusion distribution and interface length with same *A*_m_:*A*_inc_ ratios in (**a**) and (**b**).

Moreover, in the case of the same area fraction *A*_m_:*A*_p_, identical material property ratios, inclusion shapes, and interface characteristics, an increase in scattering can be achieved through inclusion arrangement (Figure 15). When determining homogenized material properties, the rule of mixtures is often applied, which considers only the volumetric (or areal) fraction of the constituent phases. The effect of both distribution and increased interface length is evident in Figure 15a, as it enhances scattering and, consequently, amplitude attenuation. In Figure 15a, one inclusion is replaced by 10 inclusions, while in both cases, the *A*_m_:*A*_inc_ ratio and other material property ratios remain the same. The increase in wave scattering results in a 3% increase in attenuation, correlating with increased interface length. In Figure 15a, the interface length is 3.2 times longer in the case of 10 inclusions. Similarly, in Figure 15b, in three cases, the *A*_m_:*A*_inc_ ratio and other material property ratios are identical. The area of a single inclusion corresponds to the area of 100 inclusions; the interface length is increased tenfold, resulting in an 11.3% increase in attenuation. Furthermore, redistributing the same 100 inclusions leads to an additional 4% increase in attenuation, as arrangements with inclusion overlap the enhanced phenomena associated with stress wave scattering.

## 4. Implications of Analysis for Stress Wave Barrier Zone Composite Inner Structure

The numerical results presented in the previous sections clearly demonstrate that the geometric shape, orientation, and distribution of reinforcement, as well as the presence of an interlayer, play a key role in the level of stress wave attenuation and the manner in which stress waves are scattered in discontinuously reinforced composites. This effect can be explained by a combination of mechanisms through which the stress wave, as a carrier of mechanical energy, propagates, changes direction, or is absorbed and scattered. When a stress wave reaches the matrix–inclusion interface, part of its mechanical energy is absorbed, and the wave is redistributed in multiple directions across the composite’s material phases. A portion of the wave is reflected back, another portion is transmitted into the interlayer and into the inclusion, and reflected waves interact with one another, leading to mutual attenuation and a reduction in overall wave amplitude.

For the creation of a highly efficient stress barrier zone under impacting load, the following are recommended:

- Hollow inclusions, as well as the presence of an interlayer, introduce multiple internal interfaces that markedly enhance the probability of interactions between the propagating stress wave and the internal topology of the composite.
- Hollow inclusions increase the amount of energy for deformation. The most significant improvement was observed for the hollow rectangular inclusion, which demonstrated a 20.6% higher attenuation than the solid rectangular configuration.
- The orientation of the inclusions relative to the loading direction, i.e., orientation parallel and perpendicular to stress wave direction, demonstrated that even for identical inclusion geometries, the attenuation can vary significantly (up to 18.5% for the hollow rectangular inclusion).
- The presence of a long and compliant interlayer between the matrix and the inclusion further enhances stress wave amplitude decrease, typically on the order of units.
- For the same inclusion area fraction, redistribution of inclusions can lead to a further increase, typically on the order of units.

The combined effect of the hollow inclusion shape, controlled orientation of inclusions, and their surface interlayer constitutes an effective design strategy for maximizing stress wave attenuation. However, it must be considered that a softer interlayer decreases the strength of the composite. If the stress barrier zone is not load-component, the decrease in strength is irrelevant.

## 5. Comparison with Experimental Data

Several experimental studies have demonstrated stress wave attenuation in polymer-based discontinuously reinforced composites, supporting the physical plausibility of our findings. In [38], the experimental study of shock wave propagation in particulate polymers demonstrates that composite topology significantly affects ultrasonic wave attenuation, confirming that microstructural design strongly influences wave dissipation.

The experimental findings in [39] reveal that elastic wave attenuation in particulate polymer composites strongly depends on particle content, size, and distribution, confirming that microstructural topology exerts a decisive influence on wave dissipation

The findings of [40] experimentally support our results, showing that the presence of a distinct interlayer, soft coating, or inclusion phase in a heterogeneous composite and tailored topology effectively reduces stress wave transmission during dynamic loading.

The experimental study in [41] shows that composites with multiple particle reinforcements have higher wave attenuation than simpler composites, supporting our result that adding a separate interlayer or inclusion phase increases stress wave attenuation and damping. Moreover, the experimental study in [41] stated that the same particles, when arranged differently, produce different wave attenuation and damping in the composite.

Furthermore, the experimental study in [42] highlighted the key role of composite topology in controlling wave dissipation by experiment.

These experimental studies collectively confirm that the mechanisms we consider—scattering, reflection, and mechanical energy redistribution due to particle shape and orientation, interlayer, and distribution —are consistent with observed behaviors in similar polymer–particle systems.

## 6. Conclusions

The presence of stress barrier zones is particularly beneficial under impulse loading, enabling rapid suppression of stress concentrations at the wave-front, uniform stress distribution, improved impact resistance, and reduced vibrations and noise. These mechanisms are directly applicable to engineering components, such as CNC machine tool frames, spindle housings, tool holders and adapters, robotic arms, and impact-loaded press, or forming machine elements, where controlling stress wave transmission is essential for improving structural reliability and operational precision.

The article analyzes stress wave propagation in discontinuously reinforced composites at the microscale. Using explicit finite element analysis, various cross-sectional shapes of the reinforcing particles and fibers—circle, ellipse, rectangle, and their hollow variants—are modeled within a polymer matrix. Simulated impulsive compressive loading generates a stress wave, and its attenuation at the wave-front over time is monitored.

The presented study provides new insights into how the microscale inner-structural arrangement of reinforcing inclusions influences the ability of discontinuously reinforced composites to scatter mechanical energy. The 2D numerical RUCs models capture the dominant scattering mechanisms for stress wave barrier zones—reflection, transmission, refraction, and interference—governing stress wave attenuation in discontinuously reinforced composites. The analysis was based on numerical modeling and the results confirm that microstructural topology plays a crucial role in the design of materials intended for dynamic loading.

From the perspective of designing a stress wave barrier zone, several recommendations can be drawn:

- Preference for hollow inclusions, which introduce multiple internal interfaces;
- Optimization of inclusion orientation and distribution with respect to the dominant direction of stress wave propagation;
- Use of soft and large interlayers in components of minor structural significance, where strength is not critical and damping and attenuation performance is prioritized.

The findings of this clearly demonstrate that the performance of a composite cannot be assessed solely on the basis of the volume fraction of its constituents; rather, the internal microscale structural topology plays a decisive role in controlling its dynamic response. This insight aligns with current directions in multiscale modeling, which highlight the governing influence of micro- and nanostructures on macroscopic behavior. Consequently, rational design strategies for dynamically loaded components should integrate not only material composition but also the tailored engineering of inclusion shape, orientation, and interfacial architecture. Such an approach opens new opportunities for enhancing damping efficiency, while preserving structural integrity in next-generation composite systems

For future research, we aim to focus on the possibilities offered by the interlayer and inclusion distribution, complement the study with 3D models for specific cases such as irregular 3D inclusion cavities, investigate the application of the stress barrier zone under laboratory and real operating conditions with experimental validation, and extend the analysis to harmonic or periodic excitation and frequency-dependent behavior.

The most important contribution of this work lies in the methodological framework, which enables a systematic investigation of the effects of inclusion shape, orientation, distribution, and the presence of interlayers on the damping properties, without the need for demanding experiments in the early stages of material development. This approach establishes a foundation for the efficient optimization of composite design and reduces the risk of failure in practical applications

Overall, it can be concluded that appropriately selected shape, orientation, and distribution of the reinforcing inclusions, as well as the presence of a large interlayer, represent an effective means of enhancing the damping capacity of discontinuously reinforced composites intended for a stress wave barrier zone without altering the material composition itself. This approach offers a promising pathway toward the design development of next-generation structural materials capable of providing improved reliability, durability, and safety in dynamically loaded systems.

## Figures and Tables

**Table 1:** Material mechanical properties of the composite phases.

	**Elastic Modulus *E* [10^3^ MPa]**	**Poisson’s Ratio *ν* [-]**	**Density** ***ρ* [kg/m^3^]**	**Material Type**
Matrix	*E*_m_ = 2.4	*ν*_m_ = 0.35	*ρ*_m_ = 1200	Homogeneous, Isotropic
Inclusion	*E*_inc_ = 4.8	*ν*_inc_ = 0.35	*ρ*_inc_ = 2400	Homogeneous, Isotropic
Interface	-	-	-	bonded
Interlayer	*E*_interlayer_ = 0.2 × *E*_m_	*ν*_interlayer_ = 0.35	*ρ*_interlayer_ = 1200	Homogeneous, Isotropic
	*E*_interlayer_ = 0.5 × *E*_m_			
	*E*_interlayer_ = 1.5 × *E*_m_			

## Data Availability

The original contributions presented in this study are included in the article. Further inquiries can be directed to the corresponding author.
